# Dynamics of supersonic microparticle impact on elastomers revealed by real–time multi–frame imaging

**DOI:** 10.1038/srep25577

**Published:** 2016-05-09

**Authors:** David Veysset, Alex J. Hsieh, Steven Kooi, Alexei A. Maznev, Kevin A. Masser, Keith A. Nelson

**Affiliations:** 1 Institute for Soldier Nanotechnologies, MIT, Cambridge, Massachusetts 02139, USA; 2 Department of Chemistry, MIT, Cambridge, Massachusetts 02139, USA; 3 U.S. Army Research Laboratory, RDRL–WMM–G, Aberdeen Proving Ground, Maryland 21005–5069, USA

## Abstract

Understanding high–velocity microparticle impact is essential for many fields, from space exploration to medicine and biology. Investigations of microscale impact have hitherto been limited to post–mortem analysis of impacted specimens, which does not provide direct information on the impact dynamics. Here we report real–time multi–frame imaging studies of the impact of 7 μm diameter glass spheres traveling at 700–900 m/s on elastomer polymers. With a poly(urethane urea) (PUU) sample, we observe a hyperelastic impact phenomenon not seen on the macroscale: a microsphere undergoes a full conformal penetration into the specimen followed by a rebound which leaves the specimen unscathed. The results challenge the established interpretation of the behaviour of elastomers under high–velocity impact.

High velocity ballistic impact phenomena range in scale from catastrophic events such as impact of asteroids on planets to impact of micron– and sub–micron–sized particles, which, while attracting less public attention, plays a large role in many areas of science and technology. In space exploration, micrometeoroids present both an object for investigation^1^ and a hazard to spacecraft^2^. In many earthly technologies microparticle impact often poses a problem by causing erosion, but can also be put to a good use, for example in powder blasting^3^ and cold spraying^4^. In medicine and biology ballistic microparticles are used for needle–free gene and drug delivery^5^. Despite the wide range of applications, the dynamics of microparticle impact remain unexplored. While macroscale projectile impact has been studied in real time using high–speed imaging techniques^6^, investigations of microscale impact have been limited to post–mortem analysis of impacted samples.

Elastomeric polymers are promising materials for ballistic impact protection both on the macroscale (for example, in armour panels^7^) and on the microscale, for example as protective coatings for helicopter rotor blades^8^ and in powder blasting^3^. Among elastomer candidates for enhanced ballistic protection, polyureas, polyurethanes, and PUUs have recently gained interest owing to their versatile dynamic response^9^,^10^,^11^,^12^. Because of the thermodynamic incompatibility between building blocks, these polymers present a complex morphology with domains having different degrees of microphase separation between hard and soft segments. Recent studies have indicated that the extent of phase–mixing strongly affects the segmental dynamics and the strain–rate hardening characteristics of PUUs^13^,^14^,^15^,^16^.

Experimental investigations of microparticle impact on elastomers are typically conducted by studying erosion resulting from a prolonged exposure to multi–particle impact (see, for example, refs 3,8,17, 18, 19). Rebound resilience^20^,^21^, incremental growth of cracks under cyclic loading^22^, viscoelastic effects^21^,^23^, and heat build–up^24^ were noted among other plausible attributes of relevance to solid particle erosion wear observed over a broad range of rubbery materials. The desire to study microparticle impact events in more detail motivated the development of a laser–based microscale ballistic test platform^25^ capable of measuring the velocity of individual particles; however, the results of the impact were still studied by post–mortem inspection^25^,^26^. While using this platform to study impact of silica microspheres on PUUs at velocities up to 1 km/s, we noticed that in many cases no damage of the specimen and no penetration of the particles took place. Even though it is not uncommon to see projectiles bounce off from rubber, we did not expect this to happen at bullet speed. In some instances, the microspheres were found resting on the specimen surface, again without any damage or penetration. It was nevertheless clear that at these impact velocities the specimen material must have undergone extreme deformations during the impact event. The need to find out what happens during the impact necessitated the addition of the real–time multi–frame imaging capability to the microballistic test platform.

## Results

**Figure 1:**
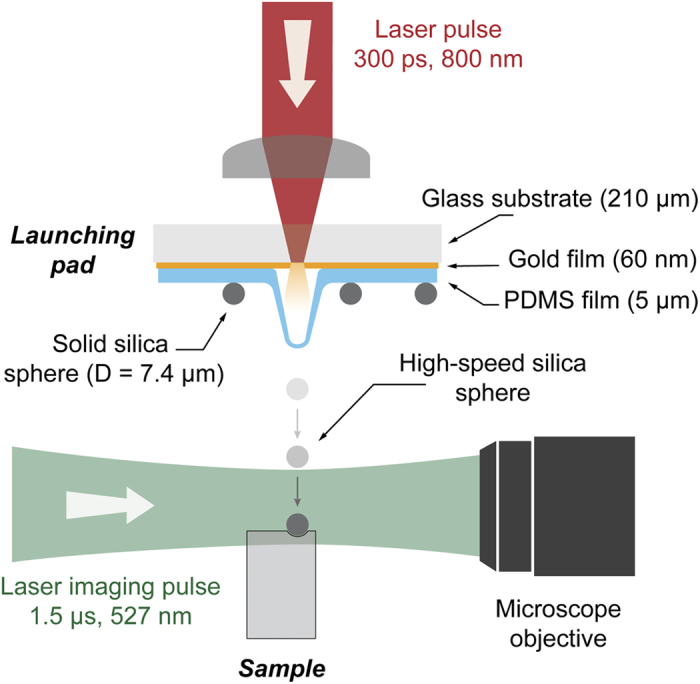
Particle launch and imaging configuration. Upon laser ablation of the gold film, the PDMS layer expands and ejects silica spheres to speeds up to 1 km/s. The sample is positioned approximately 1 mm away from the launching pad. Coming from the top, the microsphere is aimed to hit the sample near the edge facing the microscope objective. The impact is imaged in transmission using a μ s laser pulse. Multiple particles (not shown) are ejected from the launching pad on each shot. Typically, the experiment is repeated a number of times and image sequences are studied to locate impact events in the focal plane of the microscope objective.

The experimental set–up is schematically shown in Fig. 1. A laser excitation pulse was focused onto a launching pad assembly from where solid micron–sized silica spheres (*D* =  7.4 μ m ±  0.08 μ m) were ejected. Upon laser ablation of the gold film, particles were accelerated to speeds up to approximatively 1 km/s, controllable by adjusting the laser excitation pulse energy (up to 0.3 mJ). For each shot, 16 images were recorded with a high frame rate camera (SIMX 16, Specialised Imaging) using a quasi–cw laser pulse for illumination. The camera comprises 16 CCDs that can be triggered independently to record up to 16 images with exposure times as short as 3 ns. The images reported here were recorded with an exposure time of 5 ns and an inter–frame time of 35 ns. The sample was positioned so that the incoming particle hit near the edge facing the objective, as shown in Fig. 1. This was done to minimize defocusing of the image occurring as the microparticle enters the sample due to the refractive index mismatch between the air and the polymer. Multiple particle impacts in the image plane were recorded on nearly every shot. 2–3 camera CCDs were triggered prior to the impact in order to determine the instantaneous projectile velocity. (See Methods for more details.)

Elastomer specimens included PUUs of two different compositions and a cross–linked polydimethylsiloxane (PDMS). The PUU samples were composed of 4,4′ –dicyclohexylmethane diisocyanate (HMDI), diethyltoluenediamine (DETA), and poly(tetramethylene oxide) (PTMO), with a constant molar ratio of HMDI:DETA:PTMO of 2:1:1, and were fabricated with two different molecular weights, 1000 and 2000 g/mol, of the PTMO soft segment. These two PUUs were shown to exhibit different degrees of phase–mixing and correspondingly different strain hardening behaviours^13^,^14^,^15^,^16^. They will be referred to respectively as PUU1000 and PUU2000.

**Figure 2:**
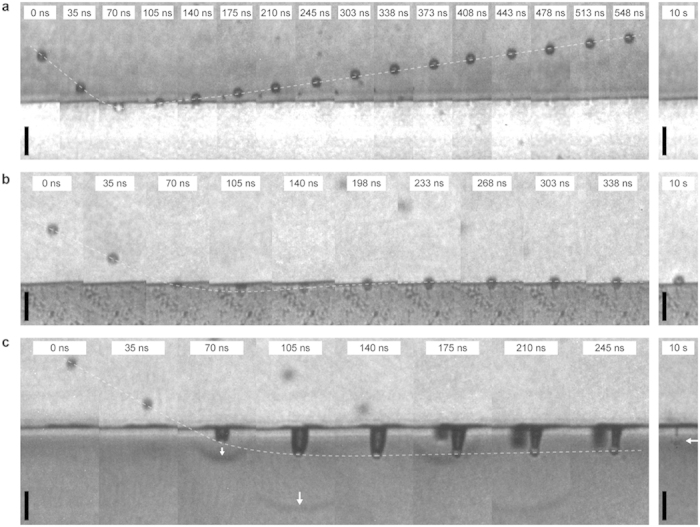
Multi–frame sequences with 5 ns exposure time showing single–projectile impacts on PUUs and PDMS. The micro–projectiles arrive from the top of the field of view. (**a**) Impact on PUU1000 at 770 m/s. The projectile penetrates conformally to about a full diameter and subsequently rebounds from the PUU surface. No permanent damage is observed in the post–mortem image (10 s). The inter–frame time is 35 ns (except between frame 8 and 9 where it is 58 ns). (**b**) Impact on PUU2000 at 670 m/s. The projectile penetrates to a depth of about 10 μ m before being pulled back to the surface of the sample, which shows no permanent damage. The inter–frame time is 35 ns (except between frame 5 and 6 where it is 58 ns). (**c**) Impact on PDMS at 940 m/s. The projectile penetrates to a maximum depth of 25 μ m before a full final embedment of 15 μ m beneath the surface (marked with a horizontal white arrow). The stress wave generated upon impact is marked by vertical white arrows in frames 3 and 4. A second particle hits the sample between frames 5 and 6. Even though it is out of focus, the particle penetration and the stress wave it generated are discernible. The inter–frame time is 35 ns. (**a**–**c**) Images are cropped from their original size to show the regions of interest (see Supplementary Videos S1–3 for full field views). The approximate projectile trajectory is marked with a white dotted line. The vertical scale bars are 20 μ m.

Figure 2 shows representative impact sequences for the three elastomer samples; the corresponding full field of view videos are available in the supplementary information (see Supplementary Videos S1–3). As shown in Fig. 2a, a micro–projectile at 770 m/s speed penetrated to a full diameter under the surface of the PUU1000 sample, with the sample surface undergoing extreme deformation to conform with the spherical projectile. The particle was then pushed out and finally rebounded with a speed of 120 m/s. Subsequently, the surface healed from the impact and showed no apparent permanent damage, as seen in the image taken a few seconds later; the absence of apparent damage at the surface was confirmed by SEM inspection. For PUU2000, an impact at 670 m/s resulted in a deeper particle penetration to about 10 microns under the surface; the particle was then pushed out and came to rest on the surface of the sample, which also showed no apparent damage. Even though no sign of post–mortem external damage was observed, internal damage or any subsequent self–healing of the PUU sample is conceivable; however, these were not investigated in this work. The behaviour of PDMS under impact was drastically different. As shown in Fig. 2c, impact on PDMS at a speed of 940 m/s resulted in deep penetration and full particle embedment, with permanent damage in the form of a channel between the particle and the surface. After penetration, the particle moved back part of the way toward the surface, so its final depth was significantly smaller than the maximum penetration.

**Figure 3:**
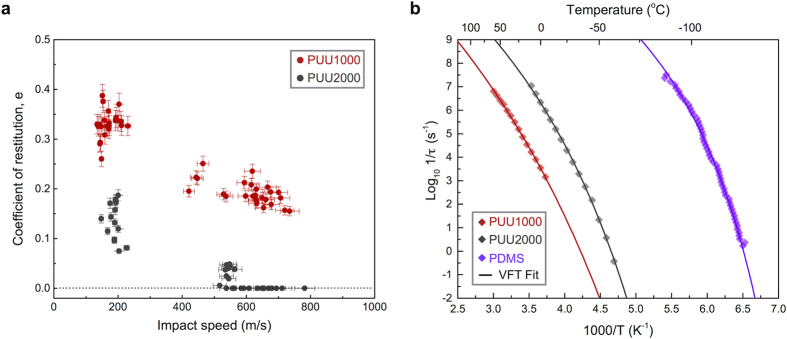
Coefficient of restitution measured for PUU1000 and PUU2000 and dielectric segmental mobility. (**a**) For PUU2000, a coefficient of restitution of zero indicates the absence of rebound and the rejection of the projectile to the surface. The error bars correspond to the uncertainty in speed calculations coming from the linear regression of particle trajectories. (**b**) Arrhenius plot of segmental mobility (1/τ ) obtained for PUU1000, PUU2000, and PDMS; solid lines are curve–fits to a Vogel Fulcher Tammann (VFT) equation.

The trends captured in Fig. 2 were observed in many impact events during multiple tests. At lower velocities, particle rebound from PUU2000 was also observed, although the rebound speed was smaller than that from PUU1000. Figure 3a shows the coefficient of restitution, defined as the ratio between the rebound velocity and the impact velocity, for PUU1000 and PUU2000 for impact speeds varying from 150 to 800 m/s. The increase of the resistance to impact from PDMS to PUU2000 to PUU1000 is correlated with the trend in the glass transition temperatures of these elastomers. PDMS has a low calorimetric glass transition temperature *T*_*g*_ (− 125 °C^27^) compared to the soft segment glass transition of PUU2000 and PUU1000 (− 64 °C and − 44 °C respectively, determined from DMA loss modulus data at 1 Hz^13^).

Since the glass transition is a dynamic phenomenon, *T*_*g*_ is strain rate and frequency dependent^28^,^29^,^30^. To further investigate the influence of molecular relaxation on the hyperelastic response, we consider the relaxation time, *τ*, or segmental mobility (*1/τ*), the reciprocal of segmental relaxation time, which is measured via broadband dielectric spectroscopy. The data shown in Fig. 3b indicate that the segmental mobility of PDMS at room temperature is significantly higher than strain rates on the order of 10^8 ^s^−1^ in the LIPIT experiment, whereas segmental mobilities of PUU2000 and PUU1000 are comparable to the strain rates; consequently PUU samples may be expected to undergo the glass transition under the LIPIT impact.

## Discussion

The responses of elastomers to macroscale high–velocity impact have been interpreted in terms of high–strain–rate induced glass transitions^7^,^10^,^31^. For example, a glassy response of polybutadiene–based polyurea characterized by brittle fracture was observed in a ballistic test at a strain rate of ~10^5 ^s^−1^ 10. Moreover, a glass transition temperature “sufficiently close to the test temperature that impact induces a transition to the glassy state”^7^ is considered a requisite property for elastomers used for ballistic protection applications.

Indeed, it would be tempting to interpret the restitution coefficient data in terms of a deformation–induced glass transition based on segmental dynamics measurements. As discussed above, the segmental mobility data shown in Fig. 3b indicates that PUU1000 could undergo a glass transition as the strain rate reaches 10^5 ^s^−1^; thus, under the impact conditions shown in Fig. 2a, PUU1000 would expect to respond in a glassy-like manner, as the strain rate at impact (~10^8 ^s^−1^) is well above the corresponding segmental mobility at ambient temperature. However, Fig. 2(a) clearly shows that PUU1000 exhibits a hyperelastic rather than glassy response, accommodating a very high level of strain without showing any sign of fracture. The conformal penetration over a full diameter of the particle implies a lower bound of 2 for the shear strain, which leads to a lower bound of ~10^8 ^s^−1^ for the strain rate, i.e. three orders of magnitude higher than strain rates cited in studies of the macroscale ballistic impact of elastomers^7^,^10^. A plausible molecular relaxation mechanism could be based on solid–state NMR measurements that suggested for any PUU composition the presence of two different populations of soft segment (SS) domains, rigid–SS and mobile–SS, with different dynamics at the molecular level^16^. At strain rate ~10^8 ^s^−1^, the mobile–SS domains could presumably transition from rubbery– to leathery–like response, while the phase–mixed rigid–SS components could undergo deformation–induced glass transition, leading to an overall hyperelastic response of PUUs under impact. Adiabatic heating upon supersonic impact could also play a role, whereby self–healing, in addition to thermal softening, facilitated by the presence of intermolecular hydrogen bonding could also be a plausible pathway toward mitigating formation of cracks and permanent damage in PUUs.

Our observations challenge the established view of the response of elastomers to high–velocity impact, according to which PUU1000 would be expected to transition to the glass–like mode of failure when impacted at ~10^8 ^s^−1^ under LIPIT based on the dielectric segmental relaxation data. We believe that the presence of multiple segmental relaxation modes may enable the hyperelastic response of hierarchical PUU elastomers, particularly over the temporal range of *μs*–*ns*. Also, relaxation measurements in the small perturbation regime (such as in dielectric spectroscopy) may be insufficient to accurately predict material behaviour under extreme strain conditions. Furthermore, the results underscore the point that the high–speed impact of microparticles is not simply a scaled–down version of macroscale ballistic impact: bullets do not bounce off rubber without damage, nor do they return to a surface after penetrating into a specimen. The capability for detailed real–time observation reveals phenomena that cannot be inferred based on post–mortem analysis. The results will provide guidance for theoretical modelling and improved fundamental understanding of microscale impact phenomena, and for the design of novel high–performance materials, through direct experimental validation. The method we have demonstrated will be applicable to a wide range of materials. The particle motion, deformation of the specimen, and stress waves in the material can be imaged with a temporal resolution of 3 ns – or better if a femtosecond pulse sequence synchronized with the camera frame rate is used to illuminate the sample. Adding polarization optics will enable imaging of stresses inside the specimen^32^. Moreover, the spectacular recent progress in time–resolved x–ray imaging^33^ indicates that the requirement of transparency could be relaxed and that nanometer–resolution imaging may be attainable. We anticipate an exciting future for studies of high–velocity impact dynamics on the micro/nanoscale.

## Methods

A 300-picosecond duration, 800-nm wavelength laser excitation pulse derived from a Ti:sapphire amplifier is focused to a spot size of about 50 μ m, using a 30-mm focal length lens, onto a launching pad assembly from which solid micron–sized silica spheres (*D* =  7.4 μ m ±  0.08 μ m) are ejected. The launching pad assembly consists of a 210 μ m thick glass substrate, a 60 nm sputter–coated gold ablation film, and a 5 μ m spin–coated PDMS layer on top of which solid silica spheres are deposited as a monolayer as described in Lee *et al.*^26^. The impact dynamics are imaged using a 10×  microscope objective, a 40-cm tube lens, and a high frame rate camera (up to 3 ×  10^8^ fps). The object plane is typically located 50 microns away from the sample front edge and the depth of focus is about 10 microns. The illumination is provided by a synchronized Q–switched Nd:YLF laser pulse (up to 1 mJ, 1.5 μ s duration, λ  =  527 nm). The PUU samples are prepared using a molar ratio of dicylcohexylmethane diisocyanate:poly(tetramethylene oxide):diethyltoluenediamine of 2:1:1, and with a poly(tetramethylene oxide) molar weight of 1000 g/mol or 2000 g/mol, following the synthesis described previously^13^. The PDMS sample is prepared using a commercial kit (Sylgard® 184) and cured for 3 hours at 60 °C. The sample is positioned relative to the launching pad and the imaging system using a 3D stage and two additional CCD cameras for live monitoring.

Regions of the polymer layer with varying particle densities can be aimed at with the laser excitation pulse. A typical shot launches between 2 and 10 particles; however not all of them hit the sample in the object plane of the imaging system as can be seen in Supplementary Videos S1–3. Typically, on the first shot after an adjustment of the excitation pulse energy, the time delay between the excitation pulse and particle impact is measured. After that the timing and locations of particle impact are sufficiently reproducible that on almost every shot, several particle impact events within the depth of focus of the object plane will be observed with 2–3 camera frames showing the particles in air prior to impact and the other frames showing the impact and subsequent dynamics.

Dielectric measurements were performed on a Novocontrol Concept 40 broadband dielectric spectrometer. Parallel–plate capacitor samples were prepared from each film. Samples were run isothermally on heating from 123 K to 333 K every 5 K. At each temperature, the samples were equilibrated for 30 minutes. The variation in temperature at each measurement temperature was less than 0.1 degrees. Measurements of the complex dielectric constant were performed from 10 MHz to 0.1 Hz under an applied potential of 1.5 Volts. At each frequency data point, a reference measurement was performed to increase accuracy.

To determine the characteristic relaxation frequencies of the dynamic glass transition and glassy state relaxation, the peak in the dielectric loss (*ε″(ω)*) associated with the relaxation was modeled with a Havriliak–Negami function^34^,^35^.


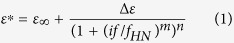


In equation (1), ε* and *ε*_∞_ are the normalized complex dielectric constant and infinite frequency dielectric constant, respectively, Δ *ε* is the dielectric strength, which is related to the dipole moment, its orientation and the number density of dipoles participating in the relaxation^36^. *m* refers to the breadth of the relaxation, *n* is the high frequency asymmetry, and *f*_*HN*_ is a characteristic frequency. In the case of glassy–state relaxations, *n* is unity. From the parameters in equation (1), the characteristic relaxation frequency (*f*_Max_) and characteristic segmental mobility (*τ*) are calculated, where the parameters listed in equation (2) are the same as those in equation (1) (see Supplementary Fig. S1 for an example of fit for PUU1000 at 25 °C).


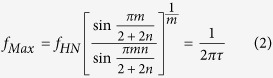


## Additional Information

**How to cite this article**: Veysset, D. *et al.* Dynamics of supersonic microparticle impact on elastomers revealed by real–time multi–frame imaging. *Sci. Rep.* **6**, 25577; doi: 10.1038/srep25577 (2016).

## Supplementary Material

Supplementary Video 1

Supplementary Video 2

Supplementary Video 3

Supplementary Information
