# Corrigendum: Dynamics of supersonic microparticle impact on elastomers revealed by real–time multi–frame imaging

**DOI:** 10.1038/srep46944

**Published:** 2018-02-16

**Authors:** David Veysset, Alex J. Hsieh, Steven Kooi, Alexei A. Maznev, Kevin A. Masser, Keith A. Nelson

Scientific Reports 6: Article number: 2557710.1038/srep25577; published online: 05 09 2016; updated: 02 16 2018

This Article contains a typographical error in the legend of Figure 2:

“The vertical scale bars are 20 μm.”

should read:

“The vertical scale bars are 24 μm.”

The calculations and results were unaffected by this error.

