# Supplementary material for: Dynamics of supersonic microparticle impact on elastomers revealed by real–time multi–frame imaging
Source: Sci Rep. 2016 May 9;6:25577. doi: 10.1038/srep25577 (PMC4860635; doi:10.1038/srep25577)
Supplement: Supplementary Information [file srep25577-s4.pdf]

Supplementary Information

Dynamics of supersonic microparticle impact on elastomers revealed by real–time multi–frame imaging

David Veysset[1,2,*], Alex J. Hsieh[1,3], Steven Kooi[1], Alexei A. Maznev[1,2], Kevin A. Masser[3], and Keith A. Nelson[1,2].

[1]*Institute for Solider Nanotechnologies, MIT, Cambridge, Massachusetts 02139, USA*

[2]*Department of Chemistry, MIT, Cambridge, Massachusetts 02139, USA*

[3]*U.S. Army Research Laboratory, RDRL–WMM–G, Aberdeen Proving Ground, Maryland 21005–5069, USA*

*Corresponding author, E-mail: dveysset@mit.edu

Supplementary notes

Speed calculations

The dimensions of the field of view were calibrated using a USAF 1951 target that was located in the object plane of the imaging system. Each 1280×960 pixels image of the video shows a field of view of 400×300 µm, with 1 µm = 3.2 pixels. The center of a particle (7.4 µm diameter), whose pixel size is about 25 pixels in diameter, is located in the image with an absolute precision of ± 2 pixels. Taking into account ± 1 pixel uncertainty in CCD alignment (calibrated with a static image), the precision on the propagation distance of the particle between two consecutive frames is ± 3 pixels, so about ± 2% over a typical propagation distance of 50 microns (lower bound). The time jitter of the camera is within ± 1 ns, set by the GHz clock (company specifications). Considering two consecutive frames separated by 35 ns, the time uncertainty is then ± 3%. Propagating the errors to the speed calculations, the uncertainty on the impact speed measurement is ± 4% and the uncertainty on the coefficient of restitution is ± 6%. Because particles are typically tracked over multiple frames and not only two consecutive ones, these uncertainties are the upper bound of the actual uncertainties. Additionally, no distortion is observed over the field of view.

Launching pad positioning

The position of the launching pad relative to the excitation pulse focusing lens (in other words, working distance) is adjusted so as to obtain a maximum particle velocity at a given laser energy. The laser spot size is estimated to be about 50 microns based on the damage inflicted to the gold coating after laser irradiation. The spot size can be tuned in order to eject more or fewer particles along with laser energy adjustments to maintain a constant laser energy density, if a similar impact speed is sought.

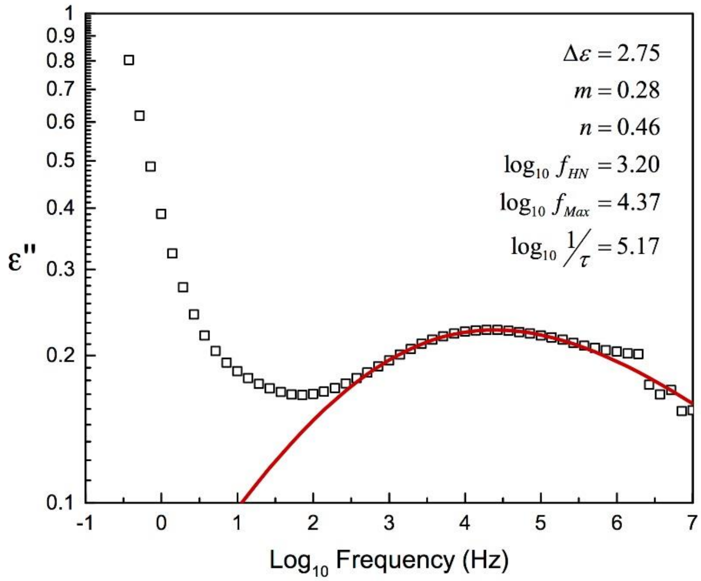

Supplementary Figure S1. Dielectric spectrum for PUU1000 at 25°C (open squares) and Havriliak–Negami function curve fit (solid line) (equation (1)). The characteristic segmental mobility (τ) is calculated using the fitted parameters $\Delta\varepsilon$, m, n, and f_{HN} in equation (2).

Supplementary Video S1. Supersonic impact video on PUU1000. Four particles travel at supersonic speed toward the sample. One particle is in focus and hits the sample's edge at a speed of 770 m/s. One out–of–focus particle misses the target and the last two particles hit the sample out of the imaging focal plane. Debris coming from the PDMS layer of the launching pad arrive during the last moments of the video. The interframe time is 35 ns (except between frame 8 and 9 where it is 58 ns), the length of the video is 548 ns and it is played at a speed of 5 frames per second. The full field of view is 400 μm × 300 μm.

Supplementary Video S2. Supersonic impact video on PUU2000. One in–focus particle hits the sample at a speed of 670 m/s while other particles are out of focus. Debris coming from the PDMS layer of the launching pad arrive during the last moments of the video. The interframe time is 35 ns (except between frame 8 and 9 where it is 58 ns), the length of the video is 548 ns and it is played at a speed of 5 fps. The full field of view is 400 μm × 300 μm.

Supplementary Video S3. Supersonic impact video on PDMS. Multiple impacts on PDMS, with one particle at 940 m/s being in focus. Other particles hitting the sample are out of focus. On the right hand–side of the field of view, one particle seems to impact the sample particularly close to the edge which would explain the different penetration pattern. Debris coming from the PDMS layer of the launching pad arrive during the

last moments of the video. The interframe time is 35 ns (except between frame 8 and 9 where it is 58 ns), the length of the video is 548 ns and it is played at a speed of 5 fps. The full field of view is 400 μm × 300 μm.
